# Calcineurin-NFAT-DSCR1.4 signaling as druggable axis in Gαq-R183Q–driven capillary malformations

**DOI:** 10.1007/s10456-026-10029-9

**Published:** 2026-02-04

**Authors:** Tong Xu, Vera Janssen, Nathalie R. Reinhard, Paula Sobrevals-Alcaraz, Robert M. van Es, Annett de Haan, Julian de Swart, Martijn Wehrens, Hannah de Kraker, Albert Wolkerstorfer, Chantal M. A. M. van der Horst, Harmjan R. Vos, Stephan Huveneers

**Affiliations:** 1 Department of Medical Biochemistry, Amsterdam Cardiovascular Sciences, Amsterdam UMC, University of Amsterdam, Meibergdreef 9, 1105 AZ Amsterdam, The Netherlands; 2 Center for Molecular Medicine, Oncode Institute, University Medical Center Utrecht, Utrecht University, 3584 CG Utrecht, The Netherlands; 3 Swammerdam Institute for Life Sciences, Section of Molecular Cytology, van Leeuwenhoek Centre for Advanced Microscopy, University of Amsterdam, Sciencepark 904, 1098 XH Amsterdam, The Netherlands; 4 Department of Dermatology, Amsterdam Institute for Immunology and Infectious Diseases, Amsterdam UMC, University of Amsterdam, Meibergdreef 9, 1105 AZ Amsterdam, The Netherlands; 5 Department of Plastic, Reconstructive, and Hand Surgery, Amsterdam Cardiovascular Sciences, Amsterdam Public Health, Amsterdam UMC, University of Amsterdam, Meibergdreef 9, 1105 AZ Amsterdam, The Netherlands

**Keywords:** Sturge-Weber syndrome, Vascular malformation, Endothelial cell, GNAQ p.R183Q, Migration, Angiogenesis, Tacrolimus, FK506

## Abstract

Capillary malformations (CMs) are congenital vascular lesions caused by somatic mutations in the *GNAQ* gene, most frequently resulting in a p.R183Q substitution in the Gαq protein in endothelial cells. However, the downstream signaling pathways by which Gαq-R183Q impairs vascular function remain poorly defined. To address this, we generated human dermal endothelial cells lacking endogenous Gαq and expressing the Gαq-R183Q mutant. Next, using SILAC-based quantitative proteomics, we mapped the Gαq-R183Q-induced endothelial phosphoproteome. These analyses identified aberrant activation of the Calcineurin–NFAT–DSCR1.4 signaling cascade as a key pathogenic feature. NFAT dysregulation and DSCR1 expression in endothelial cells were confirmed in patient-derived biopsies. Pharmacological inhibition of Calcineurin with tacrolimus partially normalized NFAT signaling in Gαq-R183Q endothelial cells. Strikingly, genetic depletion of DSCR1 in Gαq-R183Q cells fully restored Calcineurin/NFAT signaling and enabled proper endothelial migration and angiogenic sprouting, highlighting DSCR1 as a critical effector of Gαq-R183Q signaling in CMs. These findings reveal a druggable signaling circuit downstream of Gαq-R183Q that may serve as a foundation for future therapies targeting *GNAQ*-driven vascular malformations, including Sturge-Weber syndrome.

**Supplementary Information:**

The online version contains supplementary material available at 10.1007/s10456-026-10029-9.

## Introduction

Vascular malformations are a group of congenital vascular and lymphatic lesions characterized by abnormally dilated and dysfunctional vessels. Based on the type of vessel involved, they are classified into venous, arteriovenous, lymphatic, and capillary malformations. Capillary malformations (CMs) occur in approximately 3 out of every 1,000 newborns and typically present as port-wine stains, which are marked by dilated and tortuous capillaries as well as post-capillary venules [1]. A subset of patients with CM develops Sturge-Weber Syndrome (SWS), a neurocutaneous disorder in which facial port-wine stains are accompanied by ocular and leptomeningeal capillary malformations [2]. Patients with SWS exhibit vascular hypertrophy and are affected by facial disfigurement, as well as neurological and ophthalmologic complications such as seizures, epilepsy, migraines, intellectual disability, and glaucoma [1, 3, 4]. Current treatment options for SWS, including laser therapy, eye drops, and surgery to relieve intraocular pressure, are palliative rather than curative [1]. There is a clear need for better treatment strategies, and targeted molecular therapies offer a promising avenue by addressing the root causes of the vascular malformations [5]. Developing such treatments requires a thorough understanding of the molecular mechanisms behind CMs.

Both SWS and isolated port-wine stains are linked to a recurrent somatic mosaic mutation in the guanosine nucleotide-binding protein Q gene (*GNAQ*) [6–10]. This mutation results in an arginine to glutamine amino acid substitution at position 183 (p.R183Q) in the encoded Gαq protein (hereafter referred to as Gαq-R183Q) [7]. Sequencing studies have demonstrated that the Gαq-R183Q mutation is present in endothelial cells from multiple affected tissues, including: cutaneous endothelial cells of port-wine stains, brain endothelial cells in leptomeningeal malformations, and choroidal and scleral vessels in ocular lesions [8, 9, 11–15]. The Gαq protein and its close paralog Gα11 are α subunits of the heterotrimeric G protein complex, which serves as the primary signaling module downstream of G protein-coupled receptors (GPCRs). GPCRs are seven-transmembrane domain proteins that, upon binding to their cognate extracellular ligands, catalyze the activation of associated G proteins through GDP-GTP exchange on the Gα subunit [16]. Upon activation, the GTP-bound Gαq generally activates phospholipase C-beta (PLCβ), which hydrolyzes the membrane phospholipid phosphatidylinositol 4,5-bisphosphate (PIP_2_) into two major second messengers: diacylglycerol (DAG) and inositol 1,4,5-trisphosphate (IP_3_). These second messengers then initiate multiple downstream signaling cascades, with DAG activating various protein kinase C (PKC) isoforms and IP_3_ triggering calcium release from intracellular stores [17, 18]. The integrated activity of these pathways controls diverse cellular processes including growth, survival, proliferation, and migration [19, 20]. This signaling mechanism represents a widely conserved pathway across various cell types.

The specific downstream events of the Gαq-R183Q mutation in endothelial cells that contribute to CM formation are still unclear. Recent studies have demonstrated that Gαq-R183Q promotes constitutive PLCβ3 phosphorylation and subsequent activation of PKC and Calcineurin signaling in endothelial cells, resulting in transcriptional changes [21]. Also the activation of Notch and MAPK/ERK pathways have been reported in Gαq-R183Q expressing endothelial cells [20, 22], however their contribution to disease development remains incompletely understood. In fact, a systematic analysis of protein signaling differences between healthy and Gαq-R183Q expressing endothelial cells and their functional contribution to CMs is currently lacking.

To develop targeted therapies for patients with port-wine stains and SWS, it is key to identify which molecules are specifically activated downstream of Gαq-R183Q signaling and to select those that constitute druggable targets. In this study, we sought to characterize altered signaling pathways downstream of endothelial Gαq-R183Q. This requires an unbiased characterization of changes in the endothelial proteome and phosphoproteome by Gαq-R183Q, ideally in cells from patient tissues. However, the utility of primary endothelial cells from CM skin biopsies carrying somatic Gαq-R183Q mutations is limited by their finite lifespan in culture [9]. To overcome these limitations for proteomic scale studies, we established CRISPR/Cas9-mediated Gαq-knockout human dermal microvascular endothelial cell lines. These Gαq-knockout cell lines were subsequently rescued by expression of either wild-type Gαq (Gαq-WT) or mutant Gαq-R183Q. By using stable isotope labeling with amino acids in cell culture (SILAC)-based quantitative mass spectrometry, we performed comparative analyses of the phosphoproteome in Gαq-WT versus Gαq-R183Q-expressing endothelial cells. This unbiased approach identified differential protein abundance and phosphorylation levels associated with the Gαq-R183Q mutation, highlighting the importance of the Calcineurin-NFAT1/2-DSCR1.4 signaling axis in CMs. By performing functional perturbation studies we showed that inhibition of Calcineurin, or perturbation of DSCR1 expression, restores key endothelial cell functions in a Gαq-R183Q mutated background. These findings advance our understanding of the cellular and molecular mechanisms underlying CM pathogenesis and will facilitate the development of targeted therapies in CM and SWS patients.

## Results

### Characterization of Gαq knock-out HDMECs rescued with Gαq and Gαq-R183Q.

**Fig. 1:**
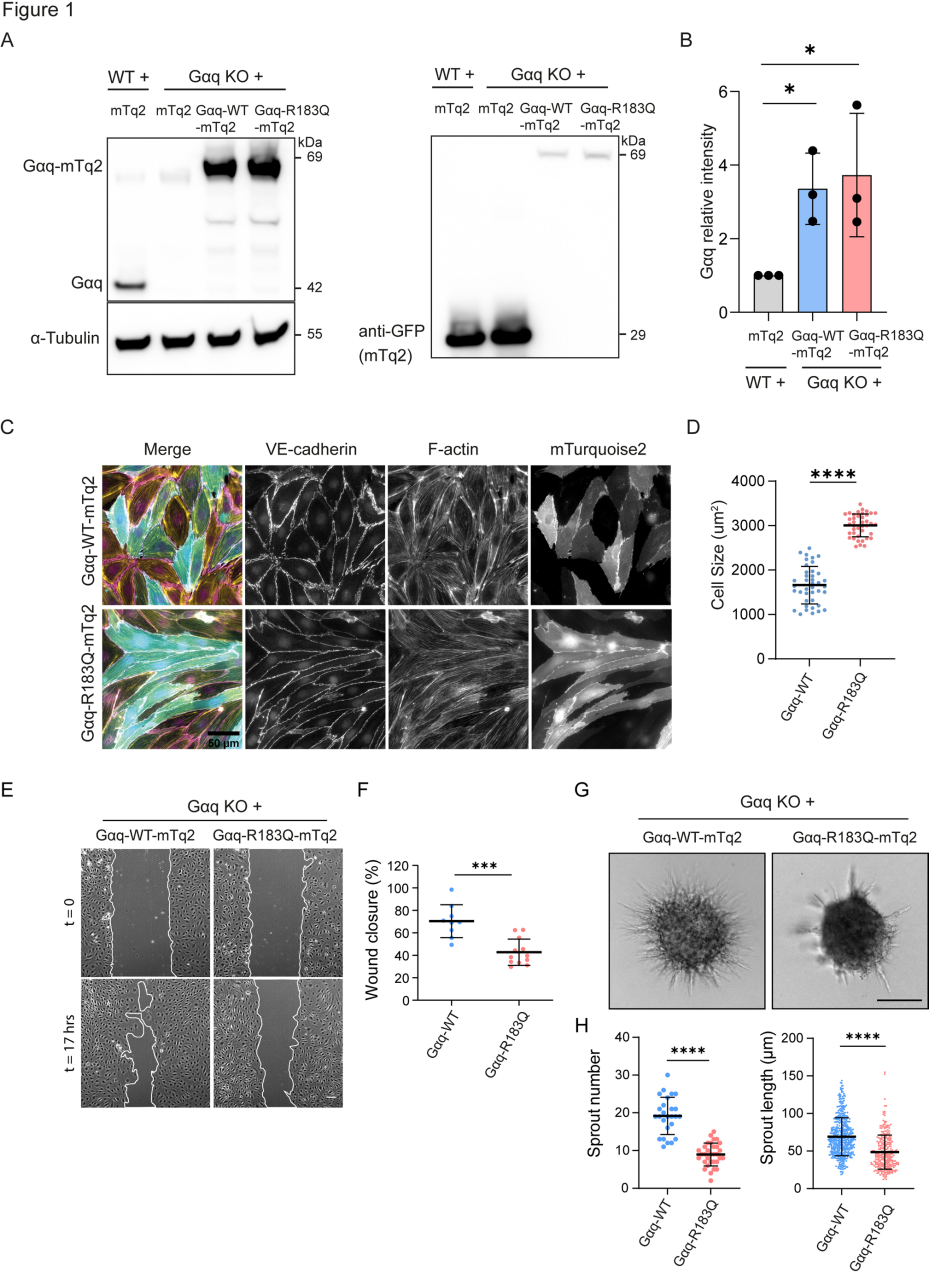
Impact of Gαq-R183Q mutation on endothelial cell morphology and function. **A** Western blot analysis of Gαq-WT-mTurquoise2 and Gαq-R183Q-mTurquoise2 re-expression in Gαq knockout (KO) human dermal microvascular endothelial cells (HDMECs). Left panel: Blots probed for Gαq and α-Tubulin (loading control). Right panel: Blots probed for GFP to detect the mTurquoise2 tag. **B** The bar plot shows quantification of Gαq band intensities. Data analyzed by two-tailed unpaired Student's t-test; mean ± SD (n = 3 independent experiments). **C** Widefield microscopy images of Gαq KO HDMECs rescued with Gαq-WT-mTurquoise2 or Gαq-R183Q-mTurquoise2. Cells were immunostained for VE-cadherin (magenta) and F-actin (yellow). Scale bar: 50 μm. **D** Cell area quantification in Gαq-WT-mTurquoise2 and Gαq-R183Q-mTurquoise2 HDMECs. Cell size for Gαq-WT mean = 1661.078 μm^2^, SD = 416.71 μm^2^, Gαq-R183Q mean = 3004.675 μm^2^, SD = 254.55 μm^2^. Data analysed by two-tailed unpaired Student's t-test; mean ± SD (n = 5 independent experiments; 40 cells in total per condition). **E** Phase-contrast time-lapse images of scratch-wound assays in Gαq KO HDMECs expressing Gαq-WT-mTurquoise2 or Gαq-R183Q-mTurquoise2 at 17 h post-scratch. White lines mark the unclosed wound area. Scale bar: 20 μm. **F** Dot plots show the percentage of remaining wound width. Data analysed by two-tailed unpaired Student's t-test; mean ± SD (n = 3 independent experiments; Gαq-WT = 9 and Gαq-R183Q = 12 samples). **G** Representative phase-contrast images of sprouting spheroids from Gαq-WT-mTurquoise2 and Gαq-R183Q-mTurquoise2 HDMECs. Scale bar: 50 μm. **H** Quantification of cumulative sprout number and length at 16 h post VEGF stimulation. Data analysed by two-tailed unpaired Student's t-test; mean ± SD (n = 3 independent experiments; for sprout number Gαq-WT = 26 spheroids and Gαq-R183Q = 32 spheroids; for sprout length Gαq-WT = 499 sprouts and Gαq-R183Q = 286 sprouts)

To investigate Gαq-R183Q specific signaling in endothelial cells, we first generated CRISPR/Cas9-mediated Gαq knock-outs (KO) from immortalized human dermal microvascular endothelial cells (HDMECs) using guide RNAs targeting the *GNAQ* gene. Next, Gαq KO HDMECs were rescued with lentiviral expression of intramolecularly mTurquoise2-tagged Gαq-wild-type or Gαq-R183Q (Fig. 1A). Western blot analysis confirmed that both rescued cell lines (hereafter referred to as Gαq-WT and Gαq-R183Q HDMECs) expressed similar Gαq levels, which were approximately three-fold higher than the endogenous protein in the parental HDMECs (Fig. 1A, B). Immunofluorescence analysis of VE-cadherin and F-actin revealed that Gαq-R183Q expression led to cell elongation, but did not affect cell–cell junctions or cytoskeletal organization when compared to Gαq-WT HDMECs (Fig. 1C). Quantitative morphometric analysis confirmed that Gαq-R183Q HDMECs exhibited larger cell sizes relative to Gαq-WT controls (Fig. 1D).

### Gαq-R183Q impairs endothelial cell migration and angiogenic sprouting.

Since dysregulated endothelial cell migration and angiogenic sprouting are drivers of vascular malformations [23, 24], we investigated how Gαq-R183Q may affect these endothelial functions. We performed scratch wound assays of monolayers formed by Gαq-WT and Gαq-R183Q HDMECs. Gαq-R183Q expression significantly impaired endothelial cell migration toward the scratch, resulting in delayed wound closure (Fig. 1E, F). To investigate the role of Gαq-R183Q in angiogenic sprouting, we conducted VEGF-induced spheroid-based sprouting assays. Sprout formation and elongation from spheroids was strongly decreased by Gαq-R183Q (Fig. 1G, H), confirming that proper Gαq signaling in ECs controls their angiogenic behavior.

### Phosphoproteomics reveals differential activation of signaling pathways in Gαq-R183Q ECs.

**Fig. 2:**
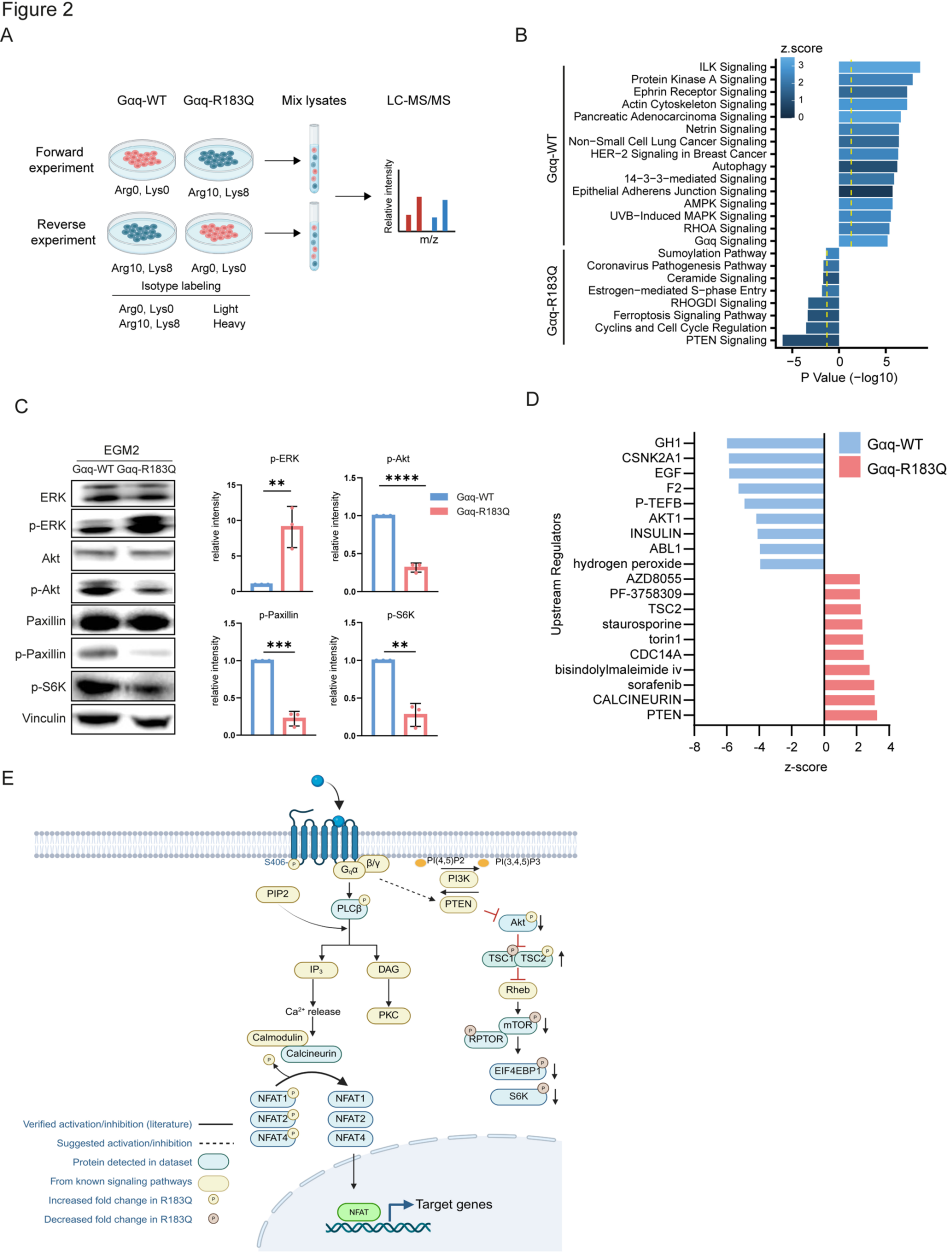
Phosphoproteomic profiling reveals differential signalling in Gαq-R183Q endothelial cells **A** Schematic of the SILAC phosphoproteomics workflow. Stable isotope labelling of amino acids in cell culture (SILAC) was performed to compare Gαq-WT and Gαq-R183Q cells. Mixed cell lysates were processed for proteomic and phosphoproteomic analysis. Schematic created with Biorender.com. **B** Ingenuity Pathway Analysis (IPA) to identify enriched signalling pathways for Gαq-WT and Gαq-R183Q. Top 15 significantly upregulated pathways for Gαq-WT and all Gαq-R183Q pathways are graphed according to their corresponding p-value and coloured according to the z-scores. Yellow dotted lines indicated thresholds with p < 0.041. **C** Western blot analysis of ERK, p-ERK, Akt, p-Akt, Paxillin, p-Paxillin, p-S6K and Vinculin (top to bottom) in lysates from Gαq-WT and Gαq-R183Q cells. Vinculin served as loading control. The bar plot shows quantification of band intensities. Data analysed by two-tailed unpaired Student's t-test; mean ± SD (n=3 independent experiments) **D** IPA of upstream regulators significantly enriched in Gαq-WT and Gαq-R183Q cells. All upstream regulators (for Gαq-WT) and top 10 significant upstream regulators (for Gαq-R183Q) are plotted by their activation z-scores and -log(p-value) thresholds (p < 0.041). **E** Proposed Gαq-R183Q signalling pathway from integrating literature and SILAC phosphoproteomics data. Schematic created with Biorender.com

To investigate how the p.R183Q mutation in Gαq changes signal transduction, we performed SILAC-based quantitative phosphoproteomic profiling. Gαq-WT and Gαq-R183Q ECs were labeled with light or heavy amino acids (Lys-0, Arg-0 or Lys-8, Arg-10, respectively) in complete endothelial culture medium, mixed post-lysis, and subjected to phosphopeptide enrichment. To address potential labeling biases, label-swap experiments, comprising forward and reverse replicates, were conducted (Fig. 2A). Phosphoproteomics on corresponding lysates identified differentially phosphorylated proteins (Suppl. Table 1) and differentially abundant proteins (Suppl. Table 2) in Gαq-R183Q ECs relative to Gαq-WT. Pathway mapping of all differentially phosphorylated sites using Ingenuity Pathway Analysis (IPA) revealed prominent upregulation of the phosphatase and tensin homolog (PTEN) pathway and downregulation of the integrin-linked kinase (ILK) pathway in Gαq-R183Q ECs (Fig. 2B). Western blot analysis of lysates from Gαq-WT and Gαq-R183Q HDMECs, showed reduced phosphorylation levels of Akt, S6K and Paxillin, confirming the detected changes in PTEN and ILK pathways (Fig. 2C). Given that previous studies reported MAPK pathway activation by Gαq-R183Q [22], while we did not detect this in our proteomic data, we evaluated ERK by Western blot analysis and found increased phosphorylated ERK levels (Fig. 2C), validating the proper function of Gαq-R183Q. To assess whether the observed effects might depend on the culture conditions, we analysed lysates from both starved and VEGF-stimulated cells. These experiments showed that the observed changes in phosphorylation on Akt, S6K, Paxillin and ERK were most prominent under VEGF-stimulated culture conditions (Suppl. Fig. S1).

Next, to identify kinases and phosphatases potentially responsible for the observed global changes in phosphorylation, we performed IPA upstream analysis on the phosphoproteomic data, which confirmed PTEN and identified Calcineurin as the top predicted regulators in Gαq-R183Q ECs (Fig. 2D). The IPA analysis pointed to reduced activation levels of multiple PTEN effectors in Gαq-R183Q ECs (Suppl. Table S3), fitting with its function as PI(3,4,5)P_3_ lipid phosphatase that antagonizes PI3K/Akt/mTOR activity. Moreover, Gαq-R183Q ECs display reduced phosphorylation levels of NFAT proteins, as well as other targets of the Calcineurin serine/threonine phosphatase (Suppl. Table S4). Taken together, the SILAC-based phosphoproteomic profiling of Gαq-R183Q ECs revealed prominent PTEN and Calcineurin activation as well as downregulation of the ILK pathway, implicating several altered signaling routes in *GNAQ*-related CMs (Fig. 2E).

### Altered phosphorylation, nuclear translocation and transcriptional activity of NFAT1/2 in Gαq-R183Q ECs.

**Fig. 3:**
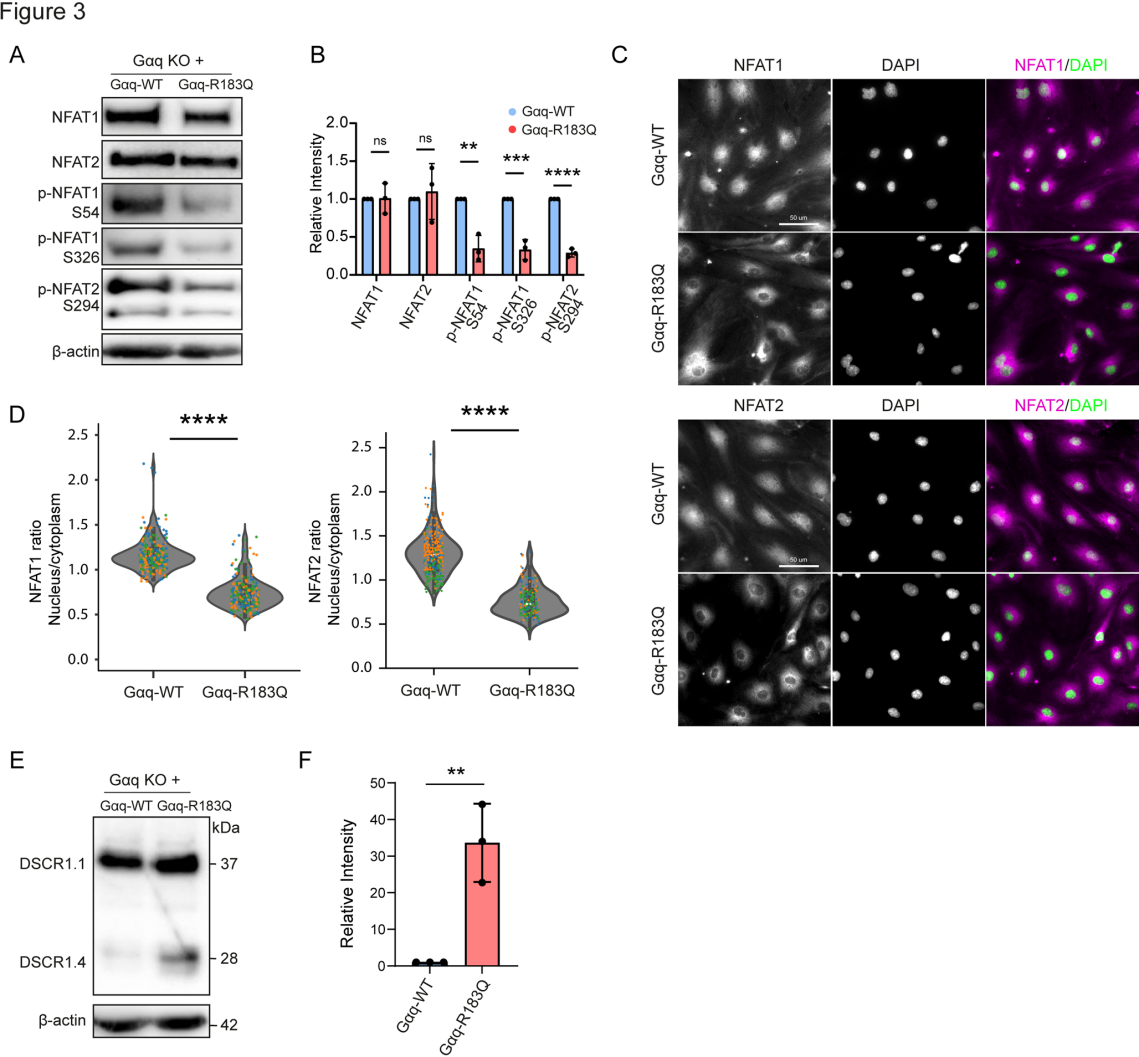
Altered phosphorylation, nuclear translocation and transcriptional activity of NFAT1/2 in Gαq-R183Q ECs. **A** Western blot analysis of NFAT1, NFAT2, NFAT1-S54, NFAT1-S326, NFAT2-S294 and β-actin (top to bottom) in Gαq-WT and Gαq-R183Q cells. β-actin served as loading control. **B** The bar plot shows quantification of band intensities. Data analysed by two-tailed unpaired Student's t-test; mean ± SD (n = 3 independent experiments). **C** Immunofluorescence images of Gαq-WT and Gαq-R183Q cells stained for NFAT1 (magenta, top panel), NFAT2 (magenta, bottom panel), and DAPI (green; nuclei). Scale bar: 50 μm. **D** Violin plots showing quantification analysis of the nuclear-to-cytoplasmic ratio of NFAT1/2 fluorescence intensity using automated image processing and segmentation. Data analyzed by two-tailed unpaired Student's t-test (n = 5 independent experiments; Gαq-WT = 177 cells and Gαq-R183Q = 182 cells for NFAT1; Gαq-WT = 317 cells and Gαq-R183Q = 344 cells for NFAT2). **E** Western blot analysis of DSCR1.1 and DSCR1.4 expression in Gαq-WT and Gαq-R183Q cells. β-actin was used as loading control. **F** Data analysed by two-tailed unpaired Student's t-test; mean ± SD (n = 3 independent experiments)

Because activation of the PLCβ-IP_3_-Calcineurin pathway was a key result from the phosphoproteomics, and has previously been associated with Gαq-R183Q-driven CMs [25], while the importance of its downstream signaling for endothelial function remains incompletely understood, we next investigated Calcineurin-NFAT regulation in Gαq-R183Q ECs in more detail. Generally, NFAT1 and NFAT2 dephosphorylation by Calcineurin induces their nuclear translocation regulating gene transcription [26–28]. The phosphoproteomics showed reduced phosphorylation levels at multiple distinct sites on the NFAT1 and NFAT2 proteins in Gαq-R183Q ECs (Suppl. Table S1). To validate these results, we performed Western blot analysis of phosphorylated Ser54 and Ser326 of NFAT1 and Ser294 of NFAT2. Indeed, phosphorylation levels of NFAT1-S54, NFAT1-S326, and NFAT2-S294 were significantly reduced in Gαq-R183Q cells (Fig. 3A, B). Both the SILAC and Western analyses showed no difference in the abundance of total NFAT1 or NFAT2 expression in Gαq-WT and Gαq-R183Q ECs (Fig. 3A, B; Suppl. Table S2). Intriguingly, immunofluorescence imaging experiments demonstrated that both NFAT1 and NFAT2 proteins remain in the cytoplasm of Gαq-R183Q ECs and fail to translocate to the nucleus like in Gαq-WT ECs (Fig. 3C). Automated quantitative analysis confirmed a strong decrease in nuclear-to-cytoplasmic ratio for both NFAT1 and NFAT2 in Gαq-R183Q cells (Fig. 3D). These results show that NFAT1 and NFAT2 proteins are constitutively dephosphorylated in Gαq-R183Q cells, while the majority of NFAT proteins remain in the cytoplasm, suggesting disrupted NFAT signaling. Next, we investigated protein expression of the NFAT target Down Syndrome Critical Region Protein 1 (DSCR1), which has been shown to be transcriptionally upregulated through overexpression of Gαq-R183Q in endothelial cells [25]. We detected an equal abundance of the canonical DSCR1.1 isoform, whereas levels of the DSCR1.4 isoform (also known as RCAN1.4), which acts as an inhibitor for NFAT [29, 30], were strongly upregulated in Gαq-R183Q ECs (Fig. 3E, F). Together, these findings reveal that Gαq-R183Q promotes Calcineurin-mediated NFAT dephosphorylation, followed by a negative feedback loop that limits nuclear NFAT levels.

### Characterization of NFAT1/2 and DSCR1 in skin capillary malformations harboring the *GNAQ* p.R183Q mutation.

**Fig. 4:**
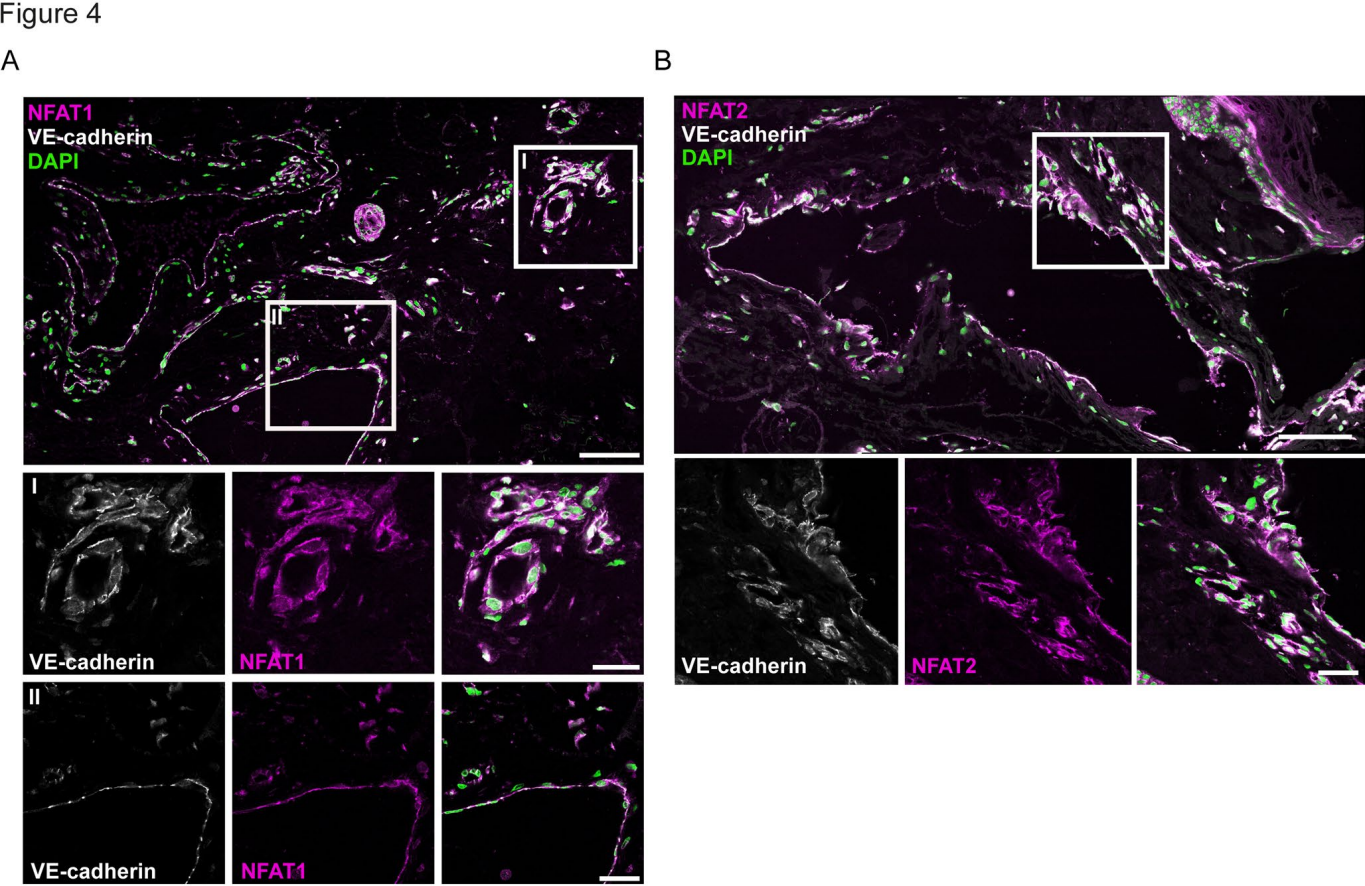
Characterization of NFAT1/2 and DSCR1 in skin capillary malformations harboring a *GNAQ* p.R183Q mutation. **A** Representative confocal images showing NFAT1 (magenta) staining in skin biopsies harboring GNAQ p.R183Q mutation. Endothelial cells were marked with VE-cadherin (white), and nuclei were stained with DAPI (green). **B** Representative images showing NFAT2 staining in skin biopsies. Endothelial cells were marked with VE-cadherin, and nuclei were stained with DAPI. Scale bars = 100 µm (upper image) and 25 µm (ROIs)

To validate whether disrupted NFAT signaling occurs within the endothelium of patients with CMs, we next performed immunofluorescence imaging of patient-derived skin biopsies that harbor the *GNAQ* p.R183Q mutation [9]. Immunostainings show DSCR1 protein expression in endothelial cells (marked by VE-cadherin) from CMs patients, as well as in epidermal epithelial cells (Suppl. Fig. S2A). Immunostainings for NFAT1/2 indicated an enriched endothelial distribution and showed that both NFAT1 and NFAT2 proteins were predominantly localized in the endothelial cytoplasm, rather than in the nucleus (Fig. 4A, B). Of note, immunostainings using phosphorylated NFAT1-S326 and NFAT2-S294 did not result in positive signal in endothelial cells in CM biopsies (Suppl. Fig. S2B). These results are consistent with the in vitro findings and suggest that NFAT signaling is disrupted in CMs.

### Inhibition of Calcineurin activity improves functions of Gαq-R183Q ECs.

**Fig. 5:**
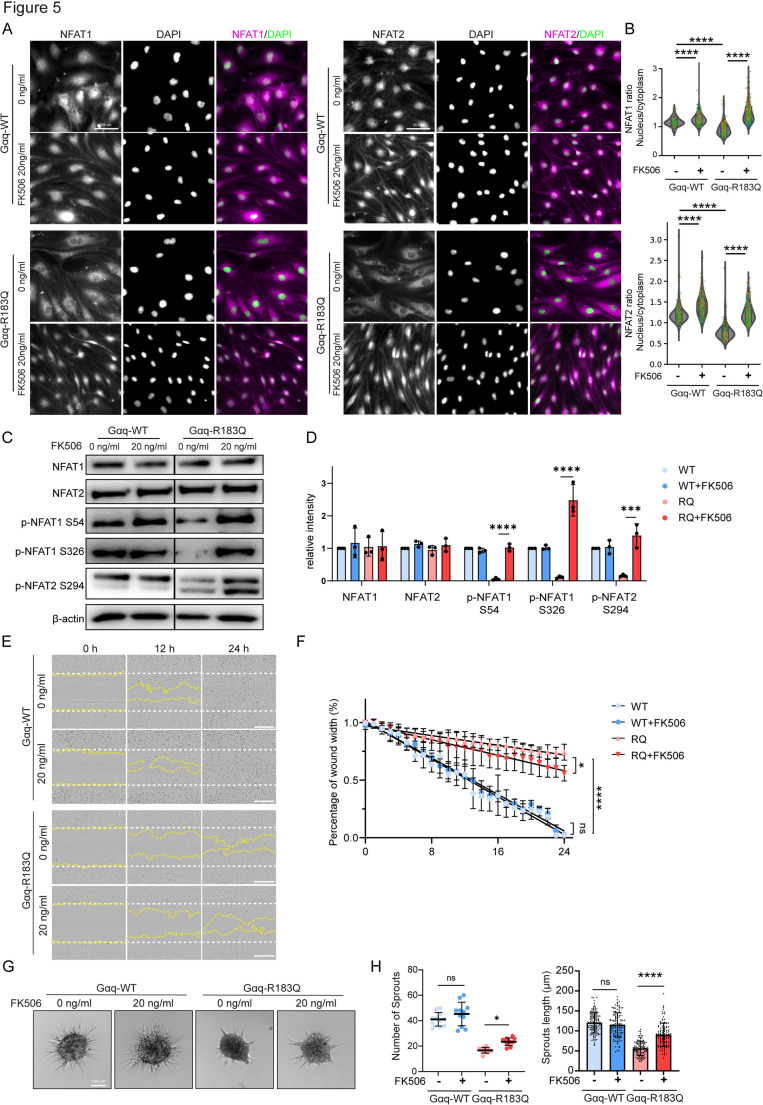
Inhibition of Calcineurin activity improves functions of Gαq-R183Q ECs. **A** Immunofluorescence analysis of Gαq-WT and Gαq-R183Q cells treated with FK506 (20 ng/ml) for 24 h, stained for NFAT1 (magenta, left panel), NFAT2 (magenta, right panel) and DAPI (green; nuclei). Scale bars: 50 μm **B** Violin plots showing quantification of the nuclear-to-cytoplasmic ratio of NFAT1/2 fluorescence intensity in Gαq-WT and Gαq-R183Q cells following FK506 treatment (20 ng/ml, 24 h) using automated image processing and segmentation. Data analysed by one-way ANOVA with Tukey’s multiple comparisons; (n = 5 independent experiments; top plots from left to right = 249, 449, 249, 328 cells for nuclear-to-cytoplasmic ratio of NFAT1, bottom plots from left to right = 269, 510, 267, 330 cells for nuclear-to-cytoplasmic ratio of NFAT2). **C** Western blot analysis of NFAT1, NFAT2, NFAT1-S54, NFAT1-S326, NFAT2-S294, and β-actin (top to bottom) in Gαq-WT and Gαq-R183Q cells treated with FK506 (20 ng/ml) for 24 h. β-actin was used as loading control. **D** Statistical analysis of immunoblot band intensities. Data analysed by one-way ANOVA with Tukey’s multiple comparisons test; mean ± SD (n = 3 independent experiments). **E** Representative phase-contrast images of Gαq-WT and Gαq-R183Q cells treated with FK506 (20 ng/ml) for 24 h in a scratch-wound assay (t = 12 and 24 h after scratch). The white dotted line indicates the initial wound area; the yellow dotted line marks the remaining unclosed wound area. Scale bars: 100 μm. **F** Quantification of wound width over time using Incucyte ZOOM™ software. Statistical comparison was based on the percentage of wound closure at t = 24 h using one-way ANOVA with Tukey’s multiple comparisons test; data are presented as mean ± SD (n = 3 independent experiments, 1 representative scratch wound per experiment). **G** Representative widefield images from spheroid-based sprouting assays of Gαq-WT and Gαq-R183Q cells treated with FK506 (20 ng/ml), 16 h post-VEGF stimulation. Scale bar: 100 μm. **H** Dot plots showing cumulative sprout length and sprout number. Statistical analysis was performed using one-way ANOVA with Tukey’s multiple comparisons test; mean ± SD (n = 3 independent experiments; plots for sprout number based on 11 spheroids per condition; for sprout length from left to right based on 126, 120, 106, 109 sprouts)

Tacrolimus (FK506) is an immunosuppressant drug that mainly works by binding to FKBP12 to inhibit Calcineurin activity [31]. To investigate the effect of FK506 on Gαq-R183Q-induced cellular events, we treated Gαq-R183Q ECs with 20 ng/ml FK506 for 24 h. The inhibition of Calcineurin by FK506 restored the nuclear-to-cytoplasmic ratio of NFAT1/2 in Gαq-R183Q ECs to WT levels (Fig. 5A, B). Strikingly, Western blot analysis showed clear FK506-mediated restoration of phosphorylation levels of NFAT1-S326, NFAT1-S54, and NFAT2-S294 specifically in Gαq-R183Q mutant cells (Fig. 5C, D). Functional assays demonstrated that FK506 slightly improved wound healing capacity (Fig. 5 E, F) and partially rescued angiogenic capacity in Gαq-R183Q ECs, as evidenced by increased sprouting number and length in spheroid-based sprouting assays (Fig. 5G, H). These findings indicate that the Calcineurin/NFAT signaling axis is important for endothelial migration and sprouting and that its dysregulation in CMs may present a potential therapeutic target.

### Depletion of DSCR1 in Gαq-R183Q ECs restores NFAT phosphorylation, endothelial migration and angiogenic sprouting.

**Fig. 6:**
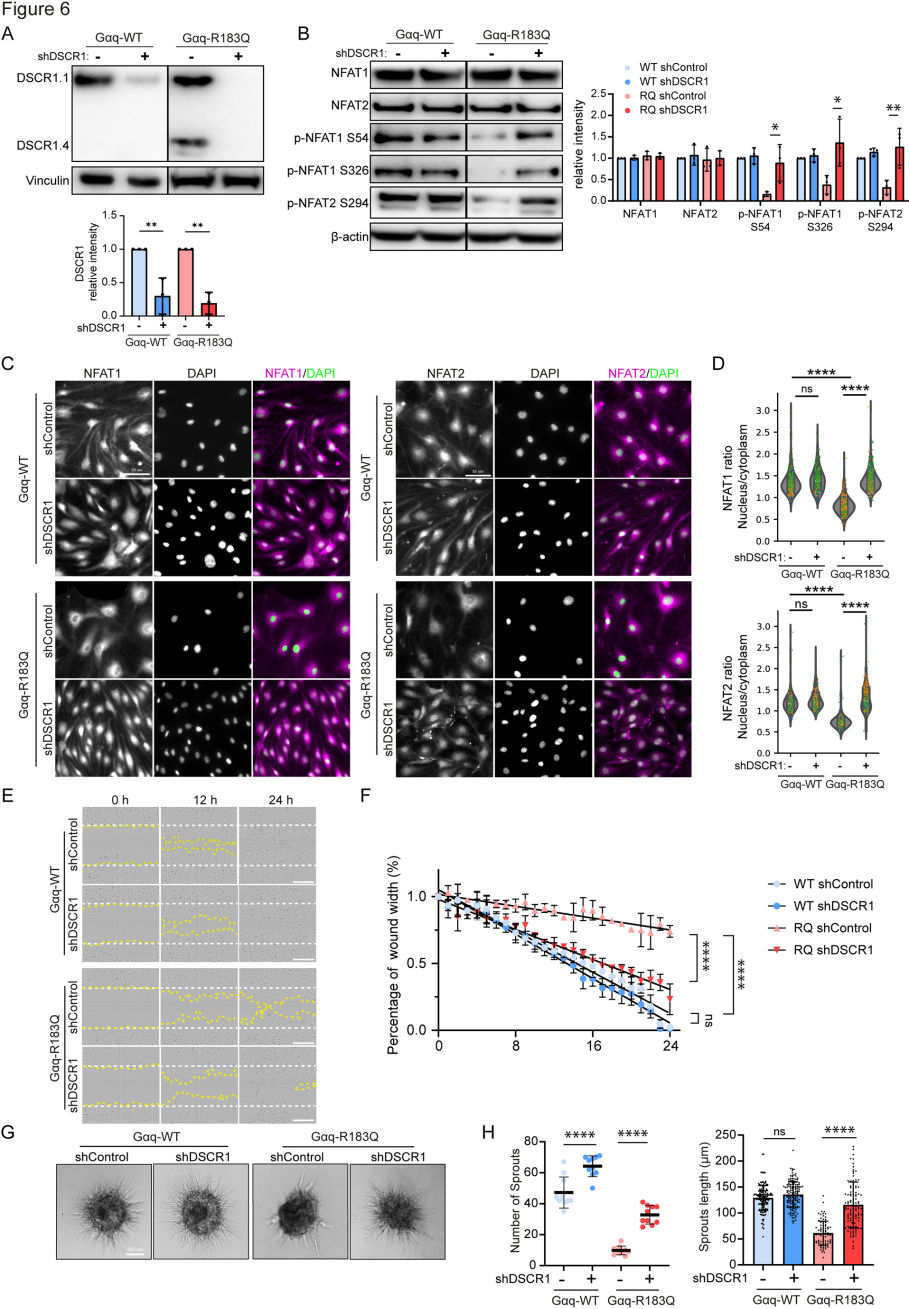
Depletion of DSCR1 in Gαq-R183Q ECs restores NFAT phosphorylation, endothelial migration and angiogenic sprouting. **A** Western blot analysis of DSCR1.1, DSCR1.4, and β-actin (top to bottom) in Gαq-WT and Gαq-R183Q cells transduced with shControl or shDSCR1. β-actin served as loading control. Data analysed by one-way ANOVA with Tukey’s multiple comparisons test; mean ± SD (n = 3 independent experiments). **B** Western blot analysis of NFAT1, NFAT2, NFAT1-S54, NFAT1-S326, NFAT2-S294 and β-actin (top to bottom) in Gαq-WT and Gαq-R183Q cells transduced with shControl or shDSCR1. β-actin served as the loading control. Graphs show statistical analysis of immunoblot band intensities. Data analysed by one-way ANOVA with Tukey’s multiple comparisons test; mean ± SD (n = 3 independent experiments). **C** Immunofluorescence images of Gαq-WT and Gαq-R183Q cells transduced with shControl or shDSCR1. Cells were stained for NFAT1 (magenta, left panel), NFAT2 (magenta, right panel), and DAPI (green; nuclei). Scale bars: 50 µm. **D** Violin plots showing quantification of nuclear-to-cytoplasmic fluorescence intensity ratios for NFAT1 (top) and NFAT2 (bottom) in the indicated cell lines. Data analysed by one-way ANOVA with Tukey’s multiple comparisons test; (n = 5 independent experiments; top plots from left to right = 276, 510, 284, 265 cells for nuclear-to-cytoplasmic ratio of NFAT1, bottom plots from left to right = 187, 229, 257, 248 cells for nuclear-to-cytoplasmic ratio of NFAT2). **E** Phase-contrast time-lapse images of scratch-wound assays in Gαq-WT and Gαq-R183Q cells transduced with shControl or shDSCR1. (t = 0, 12 and 24 h after scratch). The white dotted line shows the initial scratch area. The yellow dotted line highlight the unclosed wound area. Scale bars: 100 μm. **F** Wound width quantification over time using Incucyte ZOOM™ software. Statistical comparison was based on the percentage of wound closure at t = 24 h using one-way ANOVA with Tukey’s multiple comparisons test; data are presented as mean ± SD. (n = 3 independent experiments, 1 representative scratch wound per experiment). **G** Widefield microscopy images of spheroid sprouting assays in Gαq-WT and Gαq-R183Q cells transduced with shDSCR1, 16 h post-VEGF stimulation. Scale bar: 100 μm. **H** Dot plots showing cumulative sprout length and sprout number. Statistical analysis was performed using one-way ANOVA with Tukey’s multiple comparisons test; mean ± SD (n = 3 independent experiments; plots for sprout number based on 10 spheroids per condition; for sprout length from left to right based on 157, 149, 86, 113 sprouts)

The expression of DSCR1.4 can inhibit excessive Calcineurin/NFAT signaling [29, 30], through which it controls endothelial-driven angiogenesis and inflammatory responses [32, 33]. To assess the potential usefulness of DSCR1 as a molecular target for therapies in CMs, we next determined its importance for Gαq-R183Q-ECs. To achieve this, DSCR1 expression was depleted using lentiviral shRNA in Gαq-WT and Gαq-R183Q ECs, resulting in efficient silencing of DSCR1.1 and DSCR1.4 in Gαq-R183Q ECs (Fig. 6A). DSCR1 depletion significantly increased phosphorylation levels at NFAT1-S54, NFAT1-S326, and NFAT2-S294 in Gαq-R183Q ECs, but did not further increase their levels in Gαq-WT cells (Fig. 6B). DSCR1 knockdown promoted nuclear translocation of both NFAT1 and NFAT2 in Gαq-R183Q-expressing cells (Fig. 6C, D). Scratch wound assays demonstrated that DSCR1 depletion almost completely restored endothelial migration capacity in Gαq-R183Q ECs to that of normal ECs (Fig. 6E, F). Additionally, DSCR1 knockdowns strongly enhanced the angiogenic capacity of Gαq-R183Q ECs in spheroid-based sprouting assays (Fig. 6G, H). These results demonstrate that the depletion of DSCR1.4 from Gαq-R183Q endothelial cells relieves its negative feedback potential on the Calcineurin/NFAT pathway, leading to significantly improved endothelial functions. These findings indicate that specific pharmacological targeting of the Calcineurin-NFAT-DSCR1.4 signaling axis may respresent a promising therapeutic strategy for CMs.

## Discussion

Since the identification of the recurrent p.R183Q substitution in the GNAQ gene in SWS and nonsyndromic CMs, studies have focused on understanding its consequence for pathogenesis, signaling pathways, and endothelial dysfunction [7, 25, 34]. In this study, we employed Gαq-knockout human dermal ECs rescued with either Gαq-R183Q or Gαq-WT to systematically map differential signaling in CMs through phosphoproteomic and proteomic profiling. We present, for the first time, a comprehensive comparison of the relative phosphoproteome between Gαq-WT and Gαq-R183Q ECs. The analysis identified the PTEN- and Calcineurin-controlled pathways as prominently dysregulated in Gαq-R183Q ECs.

The Gαq-R183Q mutation thus activates multiple downstream pathways, including the canonical PLCβ pathway [35]. Activated Gαq promotes PLCβ-dependent PIP₂ hydrolysis to IP₃ and DAG, and the resulting IP₃-induced intracellular Ca^2⁺^ rise enables Calmodulin to activate Calcineurin [18, 36, 37]. Interestingly, recent work further showed that ECs expressing Gαq-R183Q or Gα11-R183C display constitutive intracellular calcium signaling, which arises from the hyperactivation of calcium-release–activated channels [38]. Following increased intracellular Ca^2⁺^, activated Calcineurin can dephosphorylate NFAT proteins to control transcription [39, 40]. Our current study highlights that there is an abundant reduction in the dephosphorylation of multiple NFAT proteins in Gαq-R183Q ECs. Previous transcriptomic studies indicated that the PLCβ/PIP_2_/IP_3_ pathway stimulates Calcineurin/NFAT activation, promoting DSCR1.4 expression in Gαq-R183Q mutated CMs [25]. While DSCR1 has been implicated in angiogenesis, tumor growth, and megakaryopoiesis via the Calcineurin/NFAT pathway [41–43], its role in Gαq-R183Q-driven CMs was still unknown. Here, we confirm that the Calcineurin-NFAT pathway is not only altered in Gαq-R183Q ECs, but it also critically deregulates endothelial functions through the increased expression of the DSCR1.4 isoform. The upregulation of DSCR1.4 in Gαq-R183Q ECs resulted in impaired nuclear import of NFAT1/2, endothelial migration and angiogenic sprouting. Since DSCR1.4 functions as an endogenous Calcineurin inhibitor [44, 45], these findings point to sustained activation of a negative feedback loop that controls NFAT in Gαq-R183Q ECs (Suppl. Fig. S3). Interestingly, repression of NFAT signaling also leads to disorganized angiogenic endothelial states in infantile hemangiomas [46], a childhood vascular tumor that is driven by a hyperactivating p.Q209L mutation in *GNAQ* [47]. While we detected NFAT/DSCR1 expression in CM patient biopsies, the absence of matched healthy control biopsies precluded a direct evaluation of their regulatory differences relative to normal skin capillaries. Future studies incorporating such controls will be essential to fully establish their significance in CM pathogenesis.

Endothelial PI3K/PTEN signaling towards Akt is well studied within the context of vascular anomalies [48–50]. Somatic pathogenic mutations in PTEN were recently shown to activate Akt/mTOR signaling in endothelial cells and drive overgrowth in vascular malformations (VMs) [51, 52]. In contrast, our data demonstrated reduced Akt/mTOR activity in Gαq-R183Q ECs. These findings suggest that the activation, and inactivation, of this signaling cascade needs to be precisely controlled for normal vascular development and function. The potential relevance of suppressed Akt/mTOR activity for Gαq-R183Q ECs, however, remains to be addressed. Another player implicated in CMs is Angiopoietin-2 (ANGPT2), which is transcriptionally upregulated in Gαq-R183Q-overexpressing ECs [20, 53] and has been proposed as a biomarker for CMs. The current phosphoproteomic analysis revealed no differential ANGPT2 phosphorylation levels in Gαq-R183Q ECs. This is likely explained by the notion that phosphoproteomics primarily detects intracellular signaling events, whereas ANGPT2 functions as a secreted protein [54].

Several FDA-approved Calcineurin inhibitors, including cyclosporin A, voclosporin, pimecrolimus, tacrolimus (FK506), and basiliximab are widely used in transplantation, autoimmune disorders, and inflammatory diseases [55, 56]. In this study, we focused on FK506 and its effects on Gαq-R183Q-mutated ECs. Our findings demonstrate that FK506 suppresses Calcineurin/NFAT signaling by restoring NFAT phosphorylation and enhancing angiogenic capacity in Gαq-R183Q CMs. However, while FK506’s Calcineurin inhibition shows therapeutic potential for mitigating NFAT-driven vascular dysfunction, its efficacy in rescuing endothelial migration and sprouting growth was less pronounced compared to the genetic depletion of DSCR1.4. This difference may result from reduced expression of FK506-binding proteins (FKBPs) in Gαq-R183Q endothelial cells. Phosphoproteomic analysis showed an approximately onefold decrease in FKBPs levels (including FKBP1A, FKBP11, and FKBP3) in mutant cells compared to controls. FKBPs play critical roles in endothelial signaling, angiogenesis, and vascular integrity by modulating NF-κB and mTOR/AKT pathways [57, 58]. Since FK506 acts through FKBPs to inhibit the Calcineurin/NFAT axis, diminished FKBP expression may affect the drug’s inhibitory efficacy. Moreover, Calcineurin inhibitors may only partially suppress the dysregulated signaling in endothelial Gαq-R183Q-induced capillary malformations, as parallel signal transduction pathways can remain active and continue to drive lesion development.

In summary, our study utilized a Gαq-R183Q-mutated in vitro model for CMs to delineate the cellular and functional consequences of this mutation in endothelial cells. We identified the Calcineurin-NFAT-DSCR1.4 signaling axis as a critically affected pathway in Gαq-R183Q CMs. This signal transduction pathway is amenable for modulation through both DSCR1.4 silencing and Calcineurin inhibitors, highlighting it as a potential therapeutic target for the development of selective therapies in CM and SWS patients. Repurposing existing drugs that target the Calcineurin pathway represents a potentially effective and cost-efficient strategy for developing new treatment options for CMs. Moreover, the presence of DSCR1.4 in capillary endothelium may serve as a potential biomarker for CMs.

## Methods

### Antibodies and reagents

The following antibodies and dyes were used for immunostaining (IF) and immunoblotting (WB) in this study. For immunofluorescence, we used Alexa Fluor 647-conjugated mouse monoclonal anti-human VE-cadherin (CD144, clone 55-7H1, BD Biosciences, Cat# 561567; 1:200), and PromoFluor-488 Phalloidin for F-actin staining (Promokine, Cat# PK-PF488P-7–01; 1:200). Alexa Fluor-594 chicken anti-rabbit (Thermo Fisher Scientific, Cat# A21442; 1:500) and Alexa Fluor-647 chicken anti-mouse (Thermo Fisher Scientific, Cat# A21463; 1:500) were used as secondary antibodies. Nuclei were stained with DAPI (Thermo Fisher Scientific, Cat# D3571, 1:1000). For immunoblotting, we used rabbit monoclonal anti-GNAQ (clone EPR17149, Abcam, Cat# ab199533; 1:1000), rabbit polyclonal anti-alpha-tubulin (Proteintech, Cat# 11224–1-AP; 1:10000), mouse monoclonal anti-GFP (clone B-2, Santa Cruz Biotechnology, Cat# sc-9996; 1:1000), rabbit polyclonal anti-pSer54-NFAT1 (Thermo Fisher Scientific, Cat# 44-944G; 1:1000), rabbit polyclonal anti-pSer326-NFAT1 (abcepta, Cat# AP61143; 1:1000), rabbit polyclonal anti-pSer294-NFAT2 (Amerigo Scientific, Cat# STJ9118; 1:1000), rabbit polyclonal anti-AKT (Cell Signaling, Cat# 9272; 1:1000), rabbit monoclonal anti-pSer473-AKT (Cell Signaling, Cat# 4060T; 1:1000), rabbit polyclonal anti-ERK (Santa Cruz, Cat# sc-153; 1:1000), rabbit monoclonal anti-pThr202/Tyr204-ERK1/2 (Cell Signaling, Cat# 4370T; 1:1000), mouse monoclonal anti-Paxillin (BD Biosciences, Cat# 610051; 1:1000), rabbit polyclonal anti-pTyr118-Paxillin (Thermo Fisher Scientific, Cat# 44-722G; 1:1000), mouse monoclonal anti-pThr389-S6K (Cell Signaling, Cat# 9206; 1:1000). Additional antibodies included anti-DSCR1 (Sigma-Aldrich, Cat# D6694; 1:1000 for WB), anti-NFAT1 (Cell Signaling Technology, Cat# 5861-T; 1:1000 for WB, 1:200 for IF), and anti-NFAT2 (Abcam, Cat# ab25916; 1:1000 for WB, 1:200 for IF). For WB secondary antibodies conjugated to horseradish peroxidase (HRP) were obtained from BioRad.

### Cell culture and plasmids

CRISPR/Cas9-mediated *GNAQ* knockouts of hTERT-immortalized human dermal microvascular endothelial cell lines (HDMEC; ATCC CRL4025) were generated as follows. Briefly, annealed gRNA-FW 5’-CACCGGGGACAAGCGGGACGCCCGC-3’ and gRNA-REV 5’-AAACGCGGGCGTCCCGCTTGTCCCC-3’ oligonucleotides were inserted into the pSpCas9(BB)-2A-GFP plasmid (Addgene #48138) and transfected into wild-type HDMECs. GFP-positive cells were isolated by fluorescence-activated cell sorting (FACS), expanded from single colonies, and cryopreserved until further use. Plasmid encoding intramolecularly mTurquoise-tagged Gαq, a validated fully functional fluorescent reporter [59], was a kind gift of Dr. Joachim Goedhart (University of Amsterdam, the Netherlands). Gαq-R183Q-mTurquoise was generated using site-directed mutagenesis. Gαq-WT-mTurquoise and Gαq-R183Q-mTurquoise inserts were cloned into a self-inactivating lentiviral pLV-CMV-ires-puro vector. Lentiviral particles were produced in HEK293T cells, which were transiently transfected with third-generation packaging constructs and the lentiviral expression vector of interest using Trans-IT LTI (Mirus). Subsequently, *GNAQ* KO HDMEC were transduced with lentivirus produced from either pLV-Gαq-WT-mTurquoise or pLV-Gαq-R183Q-mTurquoise plasmids, and selected with puromycin (Sigma, 1 µg/ml) to establish stable cell lines constitutively expressing either wild-type Gαq or mutant Gαq-R183Q. No major differences in proliferation rates were observed between the Gαq-WT and Gαq-R183Q HDMECs (Suppl. Fig. S4). shControl (shC002) lentiviral constructs and the shRNA in the lentiviral pLKO.1 backbone targeting DSCR1 (TRCN0000019844) were from the Sigma-Aldrich mission library. All cell lines were cultured on gelatin-coated dishes in Endothelial Cell Growth Medium (Promocell, Cat# C-22111) with 1% Penicillin/Streptomycin (Penicillin: 100U/mL, streptomycin: 100µg/mL) at 37 °C in a humidified atmosphere with 5% CO₂. To induce serum starvation, HDMECs were incubated in Endothelial Cell Basal Medium (EBM-2; PromoCell, C-22011) supplemented with 1% Penicillin–Streptomycin (100 U/mL penicillin, 100 µg/mL streptomycin) for approximately 12 h. After starvation, cells were stimulated with human vascular endothelial growth factor (VEGF, PeproTech; 0.5 ng/ml) for 5 min. Cells were then harvested for protein extraction and subsequent Western blot analysis.

### Cell proliferation assays

To analyze cell proliferation rates, 2 × 10^3^ Gαq-WT or Gαq-R183Q HDMECs were seeded into 96-well plates and incubated at 37 °C with 5% CO₂. Cells were incubated for 3 h in culture medium supplemented with 0.45 mg/mL MTT [3-(4,5-dimethylthiazol-2-yl)-2,5-diphenyltetrazolium bromide; Sigma-Aldrich] at the indicated time points. The resulting MTT formazan crystals were solubilized in DMSO, and optical density was measured at 570 nm using a Microplate Reader (Agilent BioTek Epoch Microplate Spectrophotometer). Background absorbance was subtracted from each data point. Experiments were performed in triplicate for each cell line.

### Immunofluorescence of cells and microscopy

For immunofluorescence staining, cells grown on fibronectin-coated coverslips were fixed in 4% paraformaldehyde (PFA) diluted in PBS supplemented with 1 mM CaCl₂ and 0.5 mM MgCl₂ (PBS + +) for 10 min at room temperature. After fixation, cells were washed twice with PBS +  + , permeabilized with 0.5% Triton X-100 in PBS for 5 min at room temperature, and washed again twice with PBS. Blocking was performed in 2% BSA in PBS. Cells were then incubated with primary antibodies against VE-cadherin and F-actin for 1 h in the dark. Following incubation, coverslips were washed with PBS and mounted onto microscope slides using Mowiol 4–88 (Calbiochem, #475904) containing DABCO (Sigma-Aldrich, D27802) as an antifade agent. Images were acquired using a Nikon Eclipse TI widefield microscope equipped with a Lumencor SOLA SE II light source, standard CFP, GFP, mCherry, or Cy5 filter cubes, a 60 × Apo TIRF oil objective (NA 1.49), and an Andor Zyla 4.2 plus sCMOS camera.

To determine nucleus-to-cytoplasm ratios a custom Python script was used (Github Repository: https://github.com/Jintram/Analysis_nucleus_cyto_ratio_static). The script segments the nuclei based on the DAPI channel (using an Otsu threshold after median filtering) and determines a narrow ring around the nucleus to characterize the cytoplasm (using image dilation). For each cell, the NFAT nucleus-to-cytoplasm ratio is calculated by dividing the mean background corrected (subtraction of mode) NFAT intensity in the nuclear region by that in the cytoplasmic ring. After automated analysis, the output was reviewed manually to remove mis-segmented cells and artifacts.

### Immunofluorescence of patient samples and microscopy

Formalin-fixed, paraffin-embedded (FFPE) CM biopsies were obtained from the Department of Dermatology at a tertiary Vascular Anomalies Center, Amsterdam University Medical Centers (Amsterdam UMC), The Netherlands. The study adhered to the Declaration of Helsinki, and written informed consent was obtained from all patients. The study was approved by the Medical Ethics Committee from the Amsterdam UMC (case number NL75128.018.20), was registered at the National Trial Register in the Netherlands on February 23, 2021 (trial identification NL9295) and its direct outcome have previously been published [9]. Sections of 4 μm thickness were mounted on microscope slides, dewaxed using xylene (#28979.294, VWR Chemicals), and rehydrated through graded alcohols to water. Antigen retrieval was performed in Tris–EDTA buffer (pH 9.0) using a microwave-based protocol. Slides were blocked in 2% BSA in PBS-Tween (#P1379, Sigma Aldrich) for 1 h. Immunofluorescence staining was conducted using primary antibodies against NFAT1 (1:100), NFAT2 (1:100), DSCR1 (1:100) and VE-cadherin (1:100). Slides were incubated with primary antibodies overnight at 4 °C, followed by two washes with PBS-Tween and incubation with secondary antibodies for 1 h at room temperature. After additional PBS-Tween washes, slides were mounted with Mowiol 4–88 containing DABCO as an antifade agent. Imaging was performed using a Leica STELLARIS 8 confocal microscope (Leica Microsystems, Wetzlar, Germany) equipped with a 63 × oil immersion objective.

### Scratch assays

For the scratch assays in Fig. 1, cells were seeded onto 12-well plates coated with 5 µg/ml fibronectin. Once confluent monolayers were established, two perpendicular scratches per well were made using a sterile 200 µl pipette tip. Detached cells were removed by washing with PBS +  + , after which the cells were cultured in EGM-2 medium. Plates were mounted on an inverted Nikon Eclipse TI microscope equipped with an Okolab cage incubator and a humidified CO₂ chamber maintained at 37 °C and 5% CO₂. Live-cell imaging was performed for 17 h at 10-min intervals using phase-contrast microscopy with a 10 × CFI Achromat DL dry objective (NA 0.25) and an Andor Zyla 4.2 plus sCMOS camera. Images were processed for display using an unsharp mask filter and adjusted for brightness and contrast in ImageJ. The wound area was quantified by measuring the scratch surface using the freehand tool in ImageJ. For experiments in Fig. 5 and 6, cells were cultured in 96-well Incucyte ImageLock plates (Sartorius, #4806) and analyzed using the Incucyte live-cell imaging system. For the migration assay, cells were seeded into the plates and grown to approximately 90% confluency. A uniform scratch was created in each well using the Incucyte WoundMaker tool. Images were captured every 30 min over a 24-h period. Image analysis was performed using the Incucyte Base Analysis Software in combination with the Incucyte Plate Map Editor.

### SILAC preparation and LC–MS/MS measurement

Gαq-KO HDMECs expressing either Gαq-WT-mTurquoise2 or Gαq-R183Q-mTurquoise2 were passaged five times in SILAC EBM-2 culture medium (Lonza), lacking arginine and lysine, and supplemented with EGM-2 BulletKit and 2% FCS. Cells were cultured in SILAC medium using either ‘heavy’ medium with isotopically labeled Arg-10 and Lys-8 or ‘light’ medium with unlabeled Arg-0 and Lys-0. Lys0 (L-Lysine•2HCl ^12^C_6_^14^N_2_, REF = 88429, Cat #WB322018, Thermofisher), Arg0 (L-Arginine•HCl ^12^C_6_^14^N_4_, REF = 88427, Cat # WA321431, Thermofisher), Lys8 (L-Lysine•HCl ^13^C_6_^15^N_2_ REF = 211603902, Cat # 211CXN-LysI-708–01, Silantes), Arg10 (L-Arginine•HCl ^13^C_6_^15^N_4,_ REF = 201603902, Cat # 201CXN-ArgI-2009–01, Silantes) were dissolved according to manufacturer instructions.

For each condition, cells were plated in two 15 cm dishes and cultured to high confluency. Cells were harvested by scraping into 1.5 mL lysis buffer containing 8 M urea (MP Biomedicals, Cat# 821527), 1 M ammonium bicarbonate (Fluka, Cat# 09830), 10 mM Tris(2-carboxyethyl)phosphine hydrochloride (TCEP) (Sigma, Cat# 75259), 40 mM 2-chloroacetamide (CAA; Sigma, Cat# C0267), 1% (v/v) phosphatase inhibitor cocktail 2 (Sigma, Cat# P5726), 1% (v/v) phosphatase inhibitor cocktail 3 (Sigma, Cat# P0044), and one tablet of EDTA-free protease inhibitor cocktail (Roche, Cat# 12273700). Lysates were immediately combined from the two dishes per condition to create label pairs, with a label swap included as a control for label dependency. Samples were sonicated to shear DNA and homogenize the lysate, then diluted fourfold with 1 M ammonium bicarbonate and digested overnight with trypsin (Worthington Biochemical Corp, Cat# LS003750) at a 50:1 protein-to-trypsin ratio, in the presence of 1 mM CaCl₂ at 37 °C on a shaker at 1000 rpm.

Digested peptides were cleaned using C18 columns (Waters, 3 CC, C18 200 mg cartridge) and eluted with 1 mL of 80% acetonitrile (ACN). For phosphopeptide enrichment, samples were acidified with trifluoroacetic acid (TFA) to a final concentration of 6%, then incubated with calcium titanate (CaTiO₃) powder (Alfa Aesar, 325 mesh) at a ratio of 10:1 (protein:powder) for 10 min at 40 °C on a shaker. Following phosphopeptide binding, the CaTiO₃ powder was washed to remove non-phosphorylated peptides. The supernatant was removed, and 1 mL of wash solution (80% ACN, 1% TFA) was added. Samples were vortexed, centrifuged (500 rpm, 30 s), and the supernatant discarded. This wash cycle was repeated three times. For elution, 300 µL of 5% ammonia solution was added to the beads, and the samples were incubated for 10 min at room temperature (RT) on a heater-shaker at 800 rpm. After centrifugation, the phosphopeptide-containing supernatant was transferred to a new 1.5 mL tube and acidified with 20 µL of 20% formic acid. Samples were further cleaned using C18 StageTips and fractionated using high-pH reverse-phase fractionation into three fractions. The samples were vacuum-dried and then resuspended in 10 µL of 0.1% formic acid. All samples were vacuum-dried. Samples were resuspended in 10 µL of 0.1% formic acid, and the first two fractions were analyzed on an Orbitrap Eclipse (Thermo Fisher Scientific) using a 240-min gradient on a self-packed 50 µm C18 column. The mass spectrometry method employed a data-dependent MS2 approach with 240,000 resolution (400–1600 m/z range), HCD fragmentation at 30% NCE, 100% AGC, dynamic injection time, a cycle time of 1 s, and a 60-s dynamic exclusion. Two FAIMS settings at −45 V and −65 V were used during acquisition.

### SILAC proteomics data analysis

Raw mass spectrometry data were analyzed using MaxQuant (version 1.6.3.4) against the complete *Homo sapiens* proteome (taxonomy ID: 9606) obtained from UniProtKB. Default MaxQuant parameters were applied, with specific settings for SILAC labeling using Arg10 and Lys8. Phosphorylation of serine, threonine, and tyrosine residues (Phospho (STY)) was included as a variable modification, in addition to methionine oxidation. Trypsin was specified as the protease, allowing up to two missed cleavages. MaxQuant output files, "proteinGroups.txt" and "Phospho (STY)Sites.txt," were used for downstream analysis in R (version 4.0.4). Proteins and phosphopeptides were initially filtered to remove contaminants, reverse sequences, and proteins identified by fewer than two unique peptides. For phosphopeptides, additional filtering was performed based on the localization score. Duplicate protein entries were resolved by merging data according to gene names and protein IDs. For each identification and sample pair, the reported normalized SILAC ratios were extracted. Ratios were log2-transformed and centered to facilitate comparative analysis (Suppl. Fig. S5). Significance thresholds were set at ± 2 standard deviations from the mean, highlighting the top 5% of significant changes. Missing values were imputed using the minimum observed ratio, adjusted by a small constant, to enable their visualization in downstream analyses. The mass spectrometry proteomics data have been deposited to the ProteomeXchange Consortium via the PRIDE [60] partner repository with the dataset identifier PXD064187. All phosphopeptides and their corresponding fold-change values were uploaded into Ingenuity Pathway Analysis (IPA, Qiagen, free trial version) to identify enriched upregulated and downregulated pathways, as well as potential upstream regulators. Z-scores and p-values were calculated in IPA using Fisher’s exact test [61]. For visualization, the top 15 significant pathways were plotted for Gαq, while all significant pathways were plotted for Gαq-R183Q. IPA graphs were generated in R.

### Sprouting angiogenesis assays

For sprouting angiogenesis assays, cells were resuspended in EGM-2 medium containing 0.1% methylcellulose (4000 cP, Sigma). To generate spheroids, 750 cells in 100 µl of methylcellulose medium were seeded into each well of a U-bottom 96-well plate and incubated overnight to allow spheroid formation. The resulting spheroids were collected, resuspended in a 1.7 mg/ml collagen type I rat tail mixture (IBIDI), and plated into glass-bottom 96-well plates, which were then incubated at 37°C to allow gel polymerization. After the collagen gel had solidified, spheroids were stimulated with 50 ng/ml VEGF (Thermo Fisher Scientific) to induce sprouting overnight, as previously described [62]. Images were acquired using the EVOS M7000 imaging system with a 10 × objective. Sprout number and length were quantified using the NeuronJ plugin in ImageJ.

#### Western blot analysis

Cells were lysed in 1X Laemmli buffer (4% SDS, 48% Tris 0.5 M pH6.8, 20% glycerol, 18% H_2_O and bromophenol blue) containing 4% β-mercaptoethanol. Lysates were boiled at 95°C for 5 min to denature proteins. Proteins were separated on 10% SDS-PAGE gels using running buffer (25 mM Tris–HCl, pH 8.3, 192 mM glycine, and 0.1% SDS), and subsequently transferred to ethanol-activated PVDF membranes via wet transfer in blotting buffer [25 mM Tris–HCl, pH 8.3, 192 mM glycine, and 20% (v/v) ethanol]. Membranes were blocked with 5% non-fat milk or BSA in Tris-buffered saline (TBS) for 1 h at room temperature. Blots were incubated overnight at 4°C with primary antibodies diluted in 5% milk or BSA in TBS containing 0.1% Tween-20 (TBST). After washing, membranes were incubated with HRP-conjugated secondary antibodies for 45 min at room temperature. Following additional washes with TBS, protein bands were detected using enhanced chemiluminescence (ECL) reagents (SuperSignal West Pico PLUS, Thermo Fisher, Cat #34580) and visualized with an ImageQuant LAS 4000 (GE Healthcare) imaging system. Band intensities were quantified using the Gel Analyzer plugin in ImageJ.

#### Statistical analysis

All data and statistical analyses were performed using GraphPad Prism version 6. Bar graphs represent mean ± standard deviation (SD), while violin plots indicate the median and quartiles. Data were assessed for normality using the D'Agostino-Pearson normality test. For data with a normal distribution, a two-tailed unpaired Student's t-test was used to compare two groups. For comparisons involving more than two groups, one-way analysis of variance (ANOVA) was performed, followed by multiple comparisons tests. The statistical specifics are indicated in the corresponding figure legends. P-values are represented by asterisks and defined as follows: ns, not significant; *P < 0.05; **P < 0.01; ***P < 0.001; ****P < 0.0001.

## Supplementary Information

Below is the link to the electronic supplementary material.

Supplementary Figure and Table legends (DOCX 15 KB)

Supplementary Table S1 (XLSX 2895 KB)

Supplementary Table S2 (XLSX 917 KB)

Supplementary Table S3 (XLS 34 KB)

Supplementary Table S4 (XLS 28 KB)

Supplementary Figures (PDF 11790 KB)

## Data Availability

Mass spectrometry proteomics data are available via ProteomeXchange with identifier PXD064187.
