# Supplementary material for: Calcineurin-NFAT-DSCR1.4 signaling as druggable axis in Gαq-R183Q–driven capillary malformations
Source: Angiogenesis. 2026 Feb 4;29(2):16. doi: 10.1007/s10456-026-10029-9 (PMC12872783; doi:10.1007/s10456-026-10029-9)
Supplement: Supplementary file 1 — Supplementary Figure and Table legends (DOCX 15 KB) [file 10456_2026_10029_MOESM1_ESM.docx]

**Supplementary Information:**

**Supplementary Figure S1. Phosphorylation levels in Gαq-WT and Gαq-R183Q endothelial cells.** (A) Western blot analysis of ERK, p-ERK, Akt, p-Akt, Paxillin, p-Paxillin, p-S6K and Vinculin (top to bottom) in starved Gαq-WT and Gαq-R183Q cells with and without VEGF stimulation. Vinculin served as loading control. Bar graphs showing quantification of band intensities. Data analysed by one-way ANOVA with Tukey’s multiple comparisons test; mean ± SD (n=3 independent experiments).

**Supplementary Figure S2. Immunofluorescence staining of skin capillary malformations harboring a *GNAQ* p.R183Q mutation.** (A) Representative images showing DSCR1 staining (magenta) in a skin biopsy. Endothelial cells in blood vessels were identified by VE-cadherin staining (white). Nuclei were stained with DAPI. (B) Representative images showing NFAT1-S326 (left) and NFAT2-S294 (right) staining in skin biopsies. Endothelial cells were marked with VE-cadherin (white), and nuclei were stained with DAPI (green). Scale bars = 100 µm (upper image) and 25 µm (ROIs).

**Supplementary Figure S3. Working model of Gαq-R183Q signaling.** The Gαq-R183Q mutation leads to the activation of PLCβ signalling. Activated PLCβ produces the second messengers IP₃ and DAG, which in turn activate Calcineurin and protein kinase C (PKC), respectively. Once the Calcineurin pathway is activated, it dephosphorylates NFAT1 and NFAT2. FK506 (Tacrolimus) forms a complex with FK506 binding proteins (FKBPs), which inhibits Calcineurin activity and thereby prevents NFAT1/2 dephosphorylation. Similarly, DSCR1.4 inhibits the Calcineurin-NFAT pathway by blocking the dephosphorylation of NFAT1/2. How the phosphorylation levels of NFAT relate to their cytoplasmic sequestration in Gαq-R183Q ECs remains an outstanding question. Schematic created with Biorender.com.

**Supplementary Figure S4. Proliferation of Gαq-WT and Gαq-R183Q endothelial cells.** Line graphs indicate results from MTT proliferations assays of the generated Gαq-WT and Gαq-R183Q cell lines. Data analysed by two-tailed unpaired Student's t-test on data of day 3; mean ± SD (n=3 independent experiments).

**Supplementary Figure S5.** Scatterplot showing the relative phosphopeptide abundance in Gαq-WT and Gαq-R183Q ECs. The x-axis represents normalized log₂ ratios from the forward experiment, while the y-axis represents normalized log₂ ratios from the reverse experiment. Statistically significant peptides (p < 0.05) are highlighted in blue.

**Supplementary Table S1.** List of differentially detected phosphorylated sites identified in the SILAC analysis of Gαq-WT and Gαq-R183Q.

**Supplementary Table S2.** List of differentially expressed proteins identified in the SILAC analysis of Gαq-WT and Gαq-R183Q.

**Supplementary Table S3.** PTEN activation associated phosphorylated proteins.

**Supplementary Table S4.** Calcineurin activation associated phosphorylated proteins.
