# Supplementary material for: Calcineurin-NFAT-DSCR1.4 signaling as druggable axis in Gαq-R183Q–driven capillary malformations
Source: Angiogenesis. 2026 Feb 4;29(2):16. doi: 10.1007/s10456-026-10029-9 (PMC12872783; doi:10.1007/s10456-026-10029-9)

Supplementary Figure S1

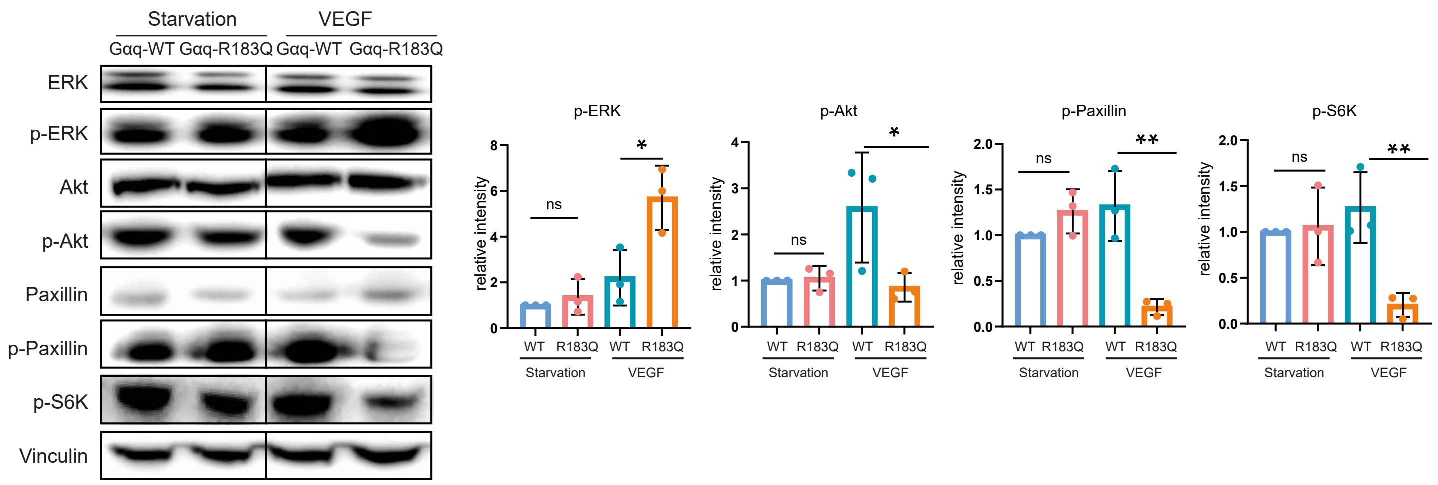

Supplementary Figure S2

A

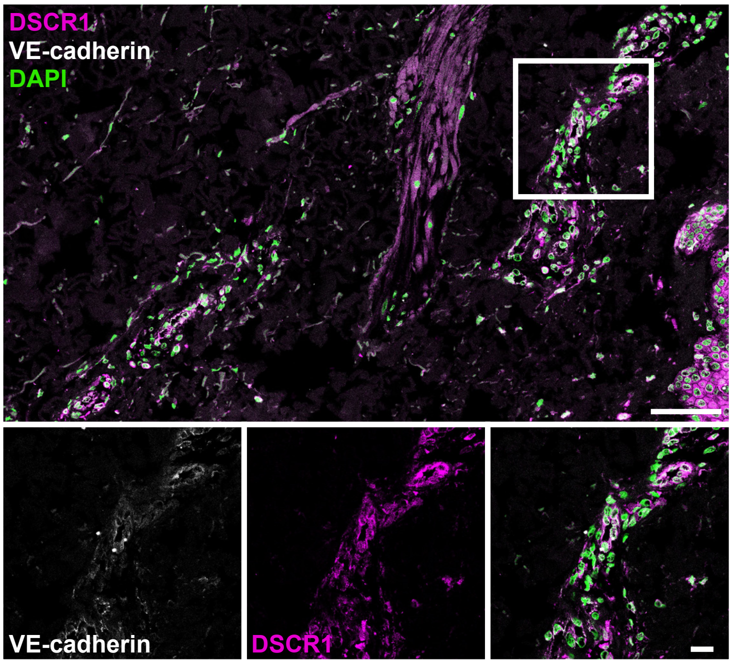

B

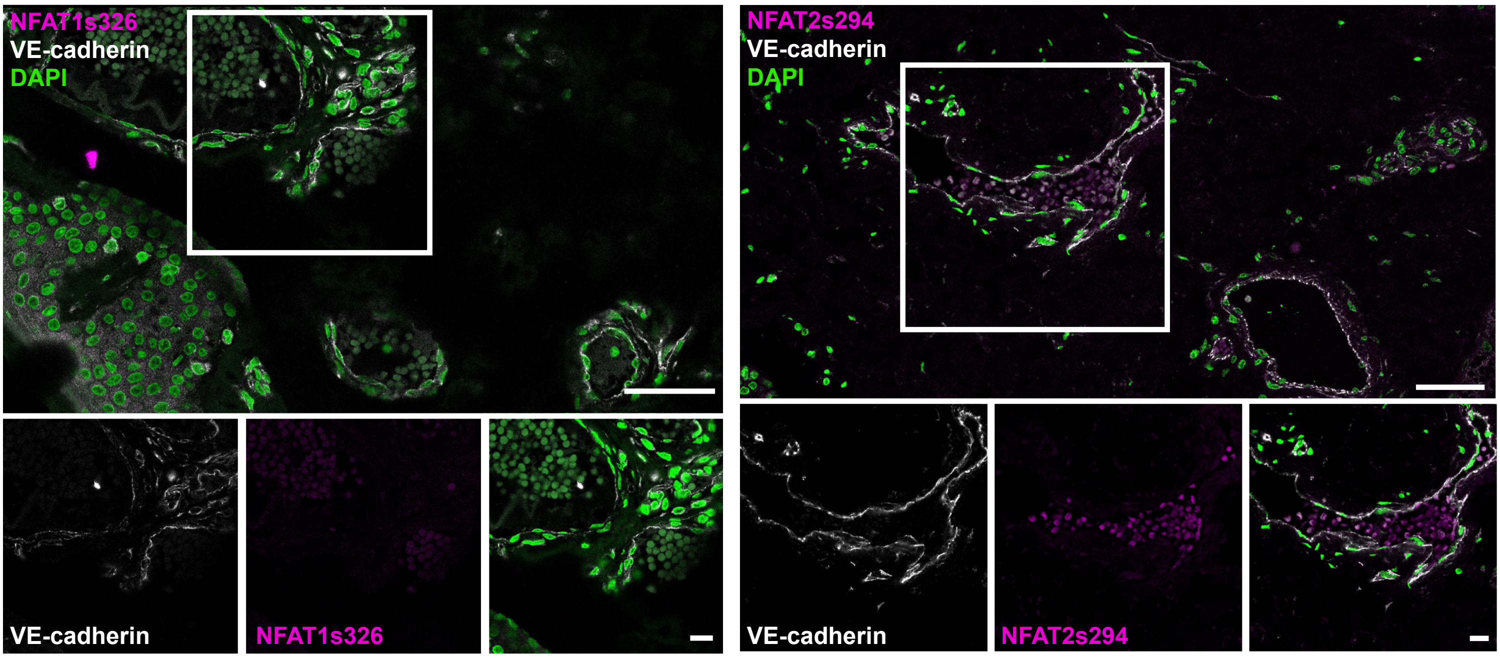

Supplementary Figure S3

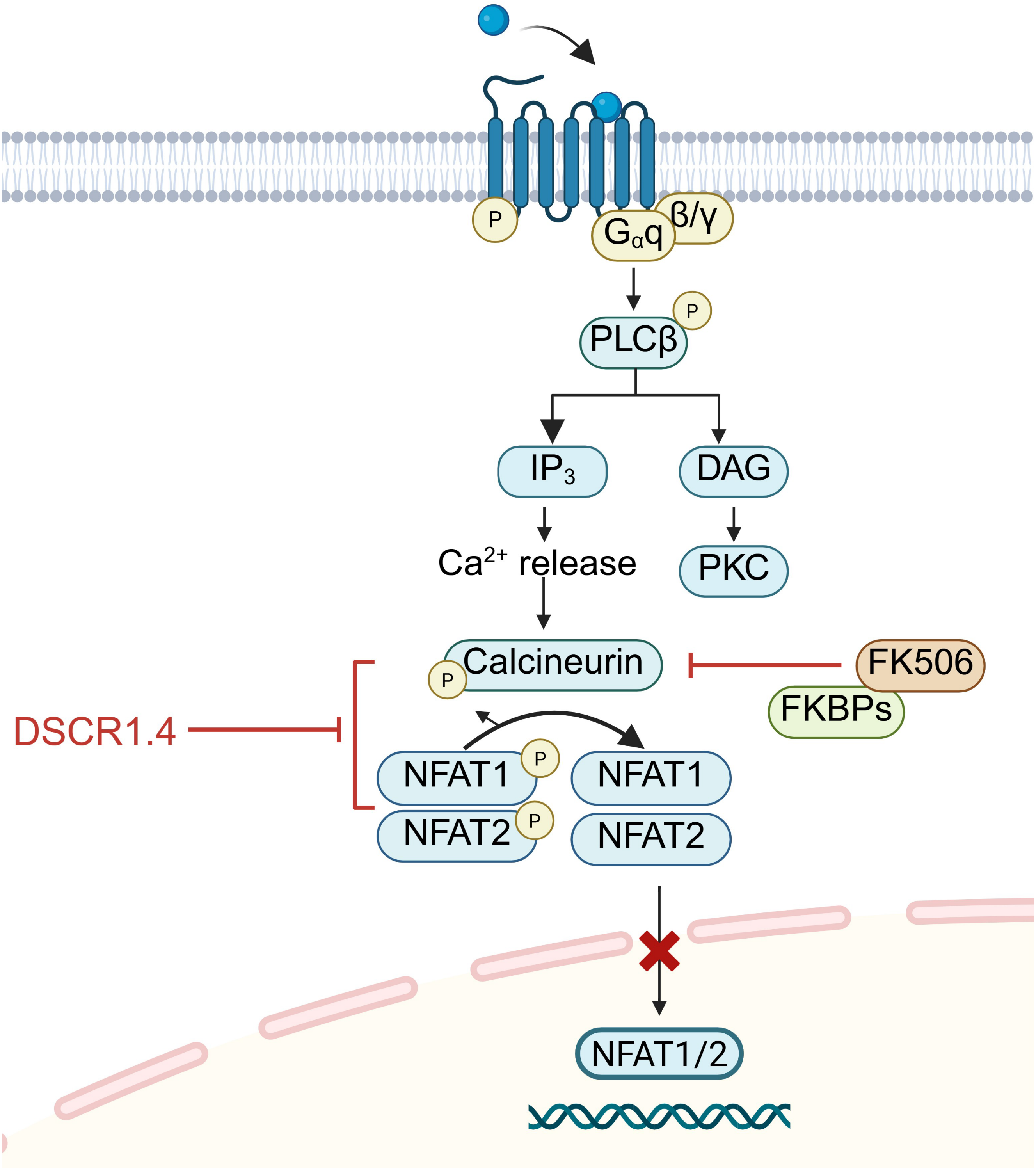

Supplementary Figure S4

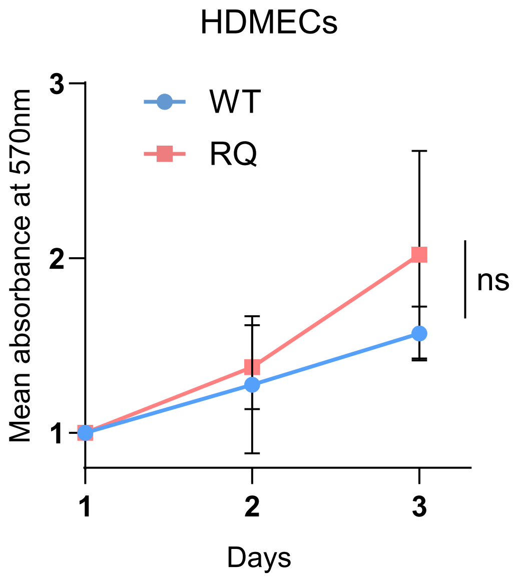

Supplementary Figure S5

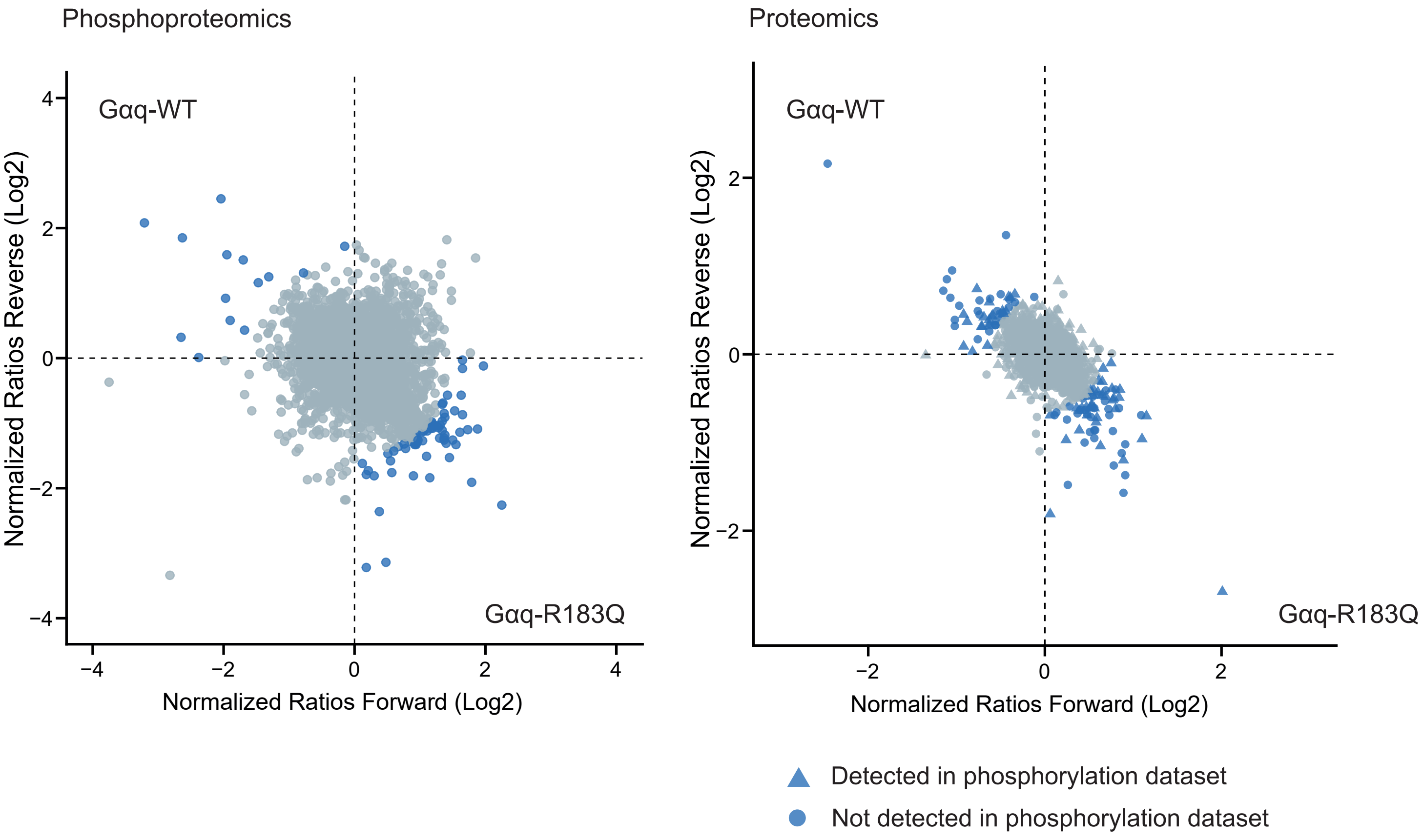

Supplement: Supplementary file 6 — Supplementary Figures (PDF 11790 KB) [file 10456_2026_10029_MOESM6_ESM.pdf]
